# Novel DNA Damage-Related Subtypes Characterization Identifies Uterine Corpus Endometrial Carcinoma (UCEC) Based on Machine Learning

**DOI:** 10.1155/2022/3588117

**Published:** 2022-08-28

**Authors:** Qi Yu, Xinqi Ge, Zheng Wang, Shu Ding, Yunfeng Jin, Liping Chen

**Affiliations:** ^1^Department of Obstetrics and Gynecology, Nantong First People's Hospital, Nantong, Jiangsu 226001, China; ^2^Department of Obstetrics and Gynecology, Affiliated Hospital of Nantong University, Nantong, China; ^3^Department of Gynecology, Obstetrics and Gynecology Hospital, Fudan University, Shanghai, China

## Abstract

**Objective:**

Accumulating evidence suggests that DNA damage is associated with numerous gynecological illnesses, particularly advanced uterine corpus endometrial carcinoma (UCEC), illustrating the involvement of the DNA damage pathway in the advancement of UCEC. This research aimed to discover a robust subtype with the potential to contribute to the scientific treatment of UCEC.

**Methods:**

In this work, the expression patterns of prognostic DNA damage-related genes were curated, and consensus clustering analyses were undertaken to determine DNA damage subtypes in patients with UCEC in the TCGA cohort. Two DNA damage-related subtypes were identified for further investigation. Differentially expressed genes (DEGs) analysis, gene ontology analysis, mutation analysis, and immune cell infraction analysis were performed to find the molecular mechanism behind it. Finally, the polymerase chain reaction (PCR) was conducted to verify the correlation of the hub genes.

**Results:**

In total, 545 patients with UCEC were tested for two distinct DNA damage subtypes. The clinical prognosis was poorer among patients with DNA damage subtype 2 than those in subtype 1. The DEGs analysis and PPI analysis showed that *ASMP*, *BUB1*, *CENPF*, *MAD2L1*, *NCAPG*, *SGO2*, and *TOP2A* were expressed higher in UCEC tissues than in the normal tissues. Immune cell infraction analysis showed that hub genes were associated with the tumor microenvironment (TME).

**Conclusion:**

Altogether, our research identified two distinct DNA damage subtypes that are complicated and heterogeneous. A better knowledge of the characteristics of the TME may be gained by quantitative measurement of DNA damage subtypes in individual patients, which can also lead to the development of more successful treatment regimens.

## 1. Introduction

Uterine corpus endometrial carcinoma (UCEC) is one of the most prevalent types of gynecologic cancers [1]. Each year, UCEC claims the lives of over 50,000 women worldwide [2]. Currently, the routine clinical therapeutic approach for patients suffering from UCEC is surgery in conjunction with radiotherapy and/or chemotherapy. Despite the fast progress in contemporary treatment, the death rate of patients with UCEC has been steadily rising over the last several years, and prognosis varies greatly from case to case [3]. Although certain clinical characteristics of patients with UCEC and many molecular biological markers have been utilized to anticipate their clinical prognosis, these strategies are associated with drawbacks [4]. Hence, the development of innovative therapy regimens and indicators is essential.

The DNA damage checkpoint is a signaling response that is triggered when there is damage to DNA [5]. Once triggered, the checkpoint is responsible for inducing several global (cell-wide) alterations in cell physiology, including cell cycle arrest, regulation of DNA replication pathways, and transcription upregulation of DNA repair genes [6]. Cancers are characterized by the presence of DNA damage, a modified regulation of DNA damage, and persistent inflammation. In spite of these restrictions, cancer cells manage to resist apoptosis and continue to proliferate. One of the hallmark features of cancer cells is their capacity to maintain proliferation and evade apoptosis while being exposed to DNA-damaging agents. Both types of cancer cells will eventually activate a DNA damage response, which will detect and repair any DNA damage that has occurred. Suppressing the process via which DNA damage is repaired gives a potential therapeutic option in both disorders.

Immunotherapy is emerging as a potentially useful treatment method since it has high specificity, long-time benefits, and few adverse effects [7]. The response rate of immune checkpoint blockade treatment is quite low in patients with UCEC due to the vast heterogeneity of the disease, which includes clinicopathologic parameters, molecular features, and immunological microenvironment [8]. Therefore, identifying the fundamental benefits in patients is essential for the advancement of immunotherapy considering UCEC variability. Despite this, the variability in the immunological environment of UCEC is not completely understood. Currently, it is difficult to arrive at a consensus signature that can be used to characterize the immunological functions in UCEC and appropriately classify patients with UCEC. Even though several prognostic signatures have been established for classifying patients with UCEC, they are unable to assess the antitumor immune activity. Hence, in this study, we examined two different DNA damage subtypes, which will lead to a more in-depth understanding of the characteristics of the TME and the discovery of a more potent prognostic biological marker for UCEC.

## 2. Materials and Methods

### 2.1. Data Resources

The Cancer Genome Atlas (TCGA) (https://tcga-data.nci.nih.gov/tcga/) database was used to extract molecular information of 545 individuals who were diagnosed with UCEC. The GDC data portal was used to retrieve the clinical data and transcriptomic profiles that were associated with the TCGA-UCEC dataset. The Ensemble IDs were converted to gene symbols, and the fragments per kilobase million (FPKM) values were converted to reflect transcripts per million (TPM).

### 2.2. Construction of DNA Damage Subtypes by Consensus Clustering Analysis

The ConsensusClusterPlus program was used to determine the DNA damage subtypes. To categorize UCEC specimens, a consensus matrix was developed with the use of consensus clustering analysis. Using PAM algorithm and Pearson correlation coefficient as a measure of distance, 500 bootstraps were displayed, each of which involved patients with UCEC included in the TCGA cohort. Moreover, the number of clusters was determined to be between 2 and 8, and consensus clustering was chosen for categorizing the relevant genes that were immunologically significant for prognosis. To determine which classification was the most accurate, a consistency matrix and a consistency cumulative distribution function were utilized [9].

### 2.3. Mutation Analysis

The TCGA dataset was searched and accessed to obtain the RNA-sequencing expression profiles, genetic mutation, and relevant clinical data of 545 patients (https://portal.gdc.com). The “maftools” package of the R software was utilized to retrieve the data on mutations, which were then visualized by this program. The histogram displays genes that had a greater mutational frequency in 545 patients.

### 2.4. Identification of DEGs

The significance analysis included within the empirical Bayes techniques that is contained inside the limma package was performed to identify DEGs. *P* value <0.01 and |logFC| > 1.5 were chosen as the threshold values to determine whether DEGs were significant. Additionally, using the cBioPortal web platform (https://www.cbioportal.org), we established a network of DEGs and their co-expression genes.

### 2.5. Analysis of Gene Ontology and Pathway Enrichment

Metascape (https://metascape.org/) is an online platform that integrates membership search, gene annotation, interactome analysis, and functional enrichment premised on over 40 separate knowledge bases via an integrated interface. Using Metascape, we carried out functional enrichment analysis of DEGs.

### 2.6. Analysis of Enrichment of Protein–Protein Interactions (PPIs)

An enrichment study of PPIs was performed using the Metascape database for each gene list that was provided. Only the physical interactions observed in STRING, with a physical score of >0.132 and BioGRID were considered. The final result is a network comprising of selected proteins that when combined with a minimum of one other member of the list creates a physical interaction. The Molecular Complex Detection (MCODE) algorithm 10 (Version 1.2; https://baderlab.org/Software/MCODE) was employed to determine which components of the network were densely connected when the number of proteins in the network ranges between 3 and 500 [10].

### 2.7. Gene Expression Validation and Survival Analysis of Hub Genes

To provide additional evidence that hub genes perform significant roles in the onset and prognosis of UCEC, we examined the expression of these genes and their value as a prognostic indicator using the GEPIA database (https://gepia.cancer-pku.cn/). GEPIA is an interactive online platform for analyzing gene expression. It comprises 9,736 samples of malignancies and 8,587 normal tissue samples [11, 12].

### 2.8. Immune Cell Infraction Analysis

To assess the reliable results of immune score evaluation, we used ssGSEA algorithms. Patients with UCEC were separated into high- and low-expression groups. To explore the role of hub genes in TME, the differences across groups were examined.

### 2.9. Quantitative RT-PCR

We extracted total RNA from paraneoplastic and tumor tissues of patients with UCEC using the TRIzol reagent (Sigma-Aldrich, St. Louis, MO, USA). Then, RNA from each sample (2 *μ*g) was subjected to quantitative reverse transcription-polymerase chain reaction using the FastStart Universal SYBR® Green Master (Roche, Germany) on an ABI QuantStudio 5 Real-Time PCR System (Thermo Fisher Scientific, USA). In a volume of reaction that was 20 *μ*l, the cDNA was utilized as a template (contained 10 *μ*l of PCR mixture, 0.5 *μ*l of reverse and forward primers, 2 *μ*l of cDNA template, and applicable volume of water). The PCR reactions were carried out as follows: the cycling conditions commenced with an initial DNA denaturation phase performed at 95°C for 30 seconds, followed by 45 cycles at 94°C for 15 s, at 56°C for 30 seconds, and at 72°C for 20 seconds. There was a triple analysis for each specimen. By employing the 2^−ΔΔCT^ method, readings from the threshold cycle (CT) were obtained and then standardized to the levels of glyceraldehyde-3-phosphate dehydrogenase (GAPDH) in all samples. The mRNA expression levels in UCEC tissues were compared to those in paracancerous tissue controls.  Table 1 presents the sequences of primer pairs corresponding to the target genes.

## 3. Results

### 3.1. Identification of Two Distinct DNA Damage Subtypes in UCEC

**Figure 1:**
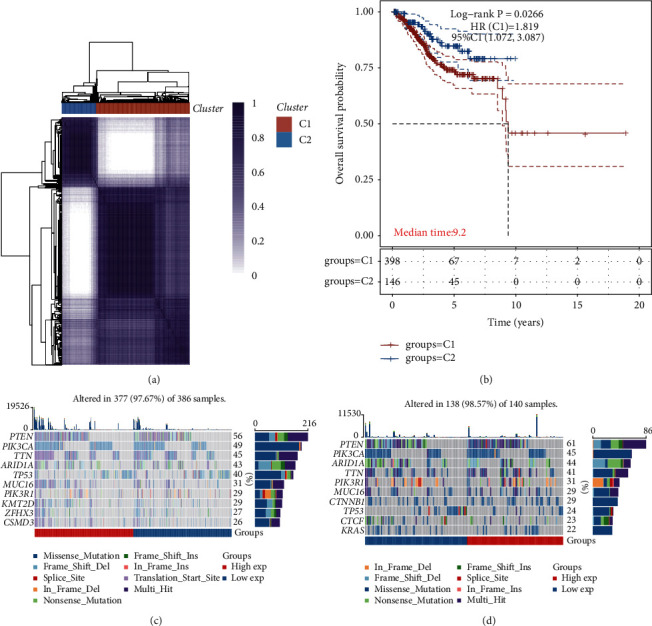
(a) A heatmap illustrating the sample clustering at consensus *k* = 2 according to the expression profiles of prognostic immune-related genes. (b) Kaplan–Meier survival analysis of the clusters. (c, d) Oncoprint illustrating the somatic mutation landscape in UCEC samples with DNA damage subtypes 1 and 2.

This research retrieved the mRNA expression profiles of hypoxia-related genes for UCEC samples from the TCGA cohort. DNA damage-related genes are listed in Table S1. Patients with UCEC were clustered according to the expression profiles of prognostic hypoxia-associated genes using consensus clustering analysis. The clustering consistency was analyzed with *k* values ranging from 2 to 8. Consequently, choosing a *k* value of 2 was the best option. This resulted in the identification of two immune subtypes among patients with UCEC, namely, immune subtype 1 (*n* = 399) and immune subtype 2 (*n* = 146) (Figure 1(a)). The survival analysis showed that the patients in subtype 2 had a poorer outcome than those in subtype 1 (Figure 1(b)).

### 3.2. Mutation Statue in Subtypes

In addition, we examined the distribution of SNPs among the UCEC samples. In all, 377 UCEC samples had genetic alterations in DNA damage subtype 1 (Figure 1(c)), whereas 161 UCEC specimens had mutations in DNA damage subtype 2 (Figure 1(d)).

### 3.3. Identification of DEGs between the Subtypes

**Figure 2:**
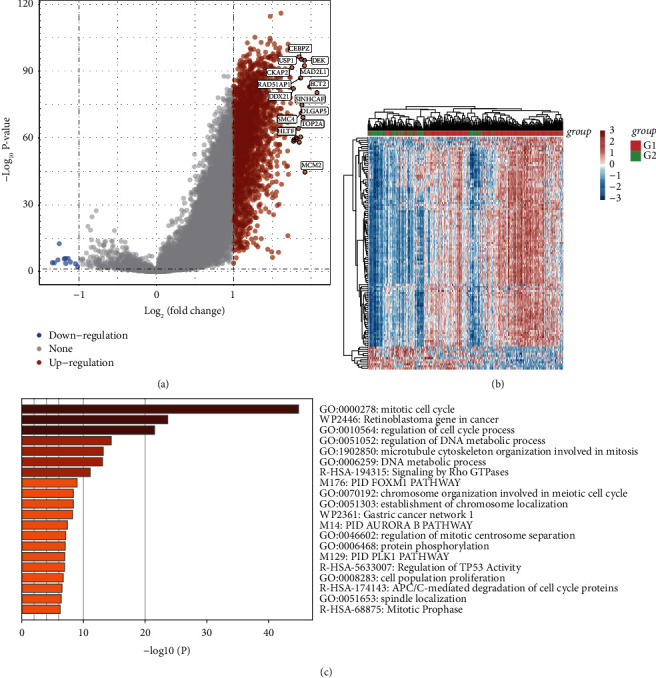
(a) The volcano plot was constructed using the fold change values and *P*-adjust. Red dots denote upregulated genes; blue dots denote downregulated genes; and gray dots denote not significant. (b) The heatmap of the differential gene expression. (c) The heatmap of enrichment analysis of DEGs.

The Limma program was used to analyze the DEGs, and the results revealed 2,465 DEGs. Of these, 2,465 genes experienced substantial upregulation, while 11 genes experienced downregulation.  Figure 2(a) displays the volcano plot created using the data from each dataset's gene expression profile.  Figure 2(b) depicts a heatmap of the top DEGs in the database.

### 3.4. Enrichment Analysis of GO terms and KEGG Pathways for DEGs

We analyzed the putative mRNA targets utilizing the gene ontology (GO) database. The analysis of the molecular function (MF), cellular component (CC), and biological process (BP) of putative targets that had been clustered utilizing the R software's ClusterProfiler program illustrated a considerable enrichment of DEGs in functions like mitotic cell cycle, retinoblastoma gene in cancer, and regulation of cell cycle process (Figure 2(c)).

### 3.5. Development of PPI Networks and Module Analysis

**Figure 3:**
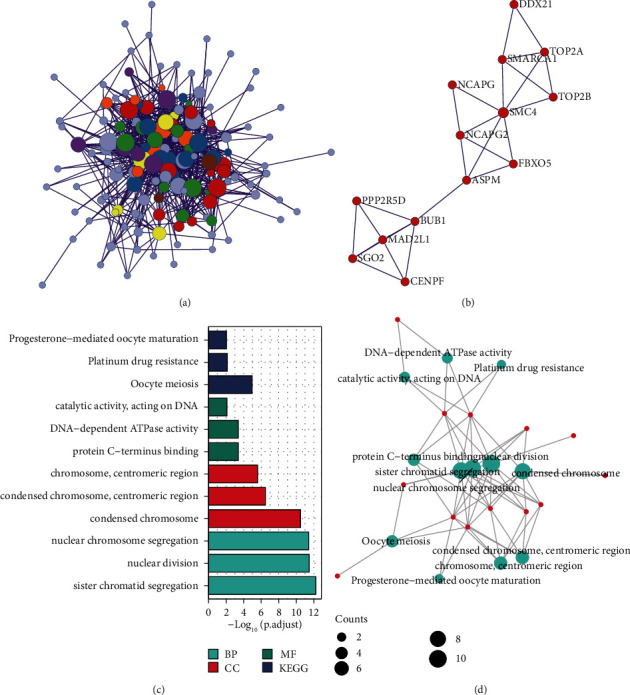
(a) The PPI network depicting DEGs and their co-expression genes. (b) Hub genes among the DEGs. (c, d) Heatmap and network of GO enrichment analysis of hub genes.

The Metascape database served as a basis for establishing a PPI network incorporating DEGs (Figures 3(a)-3(b)). The two most significant modules were extracted from this PPI network by employing MCODE, one consisting of genes that were upregulated, and the other consisting of genes that were downregulated. Hub genes were selected for further analysis. Hub genes were mostly enriched in the pathways like progesterone-mediated oocyte maturation, platinum drug resistance, and oocyte meiosis (Figures 3(c)–3(d)).

### 3.6. Analysis and Verification of Hub Genes

**Figure 4:**
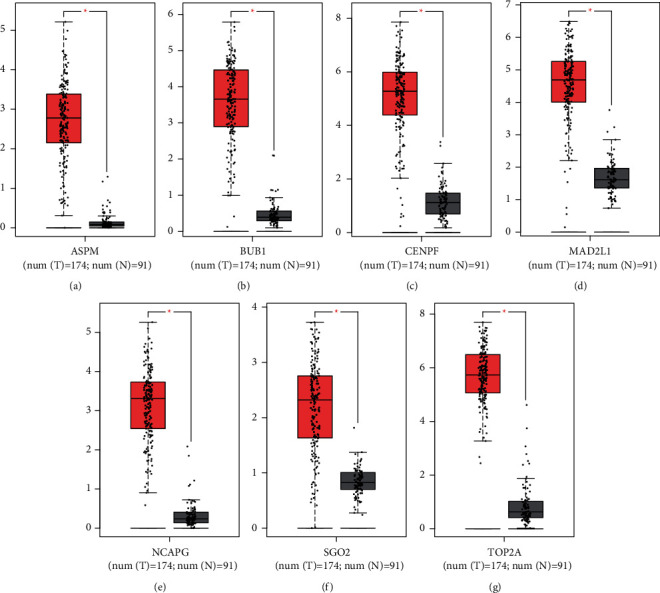
(a–g) The levels of hub gene expression and their prognostic significance based on data from the GEPIA database.

The retrieval of the GEPIA database also revealed that *ASMP*, *BUB1*, *CENPF*, *MAD2L1*, *NCAPG*, *SGO2*, and *TOP2A* had substantial differences in expression between tumor and normal tissues of UCEC (Figures 4(a)–4(g)). All hub genes had higher expression in tumor groups compared with normal groups. Moreover, the results suggested that all hub genes were valuable in UCEC, which further confirmed that the results of our study are valuable.

### 3.7. Hub Genes and Immune Infiltrates

**Figure 5:**
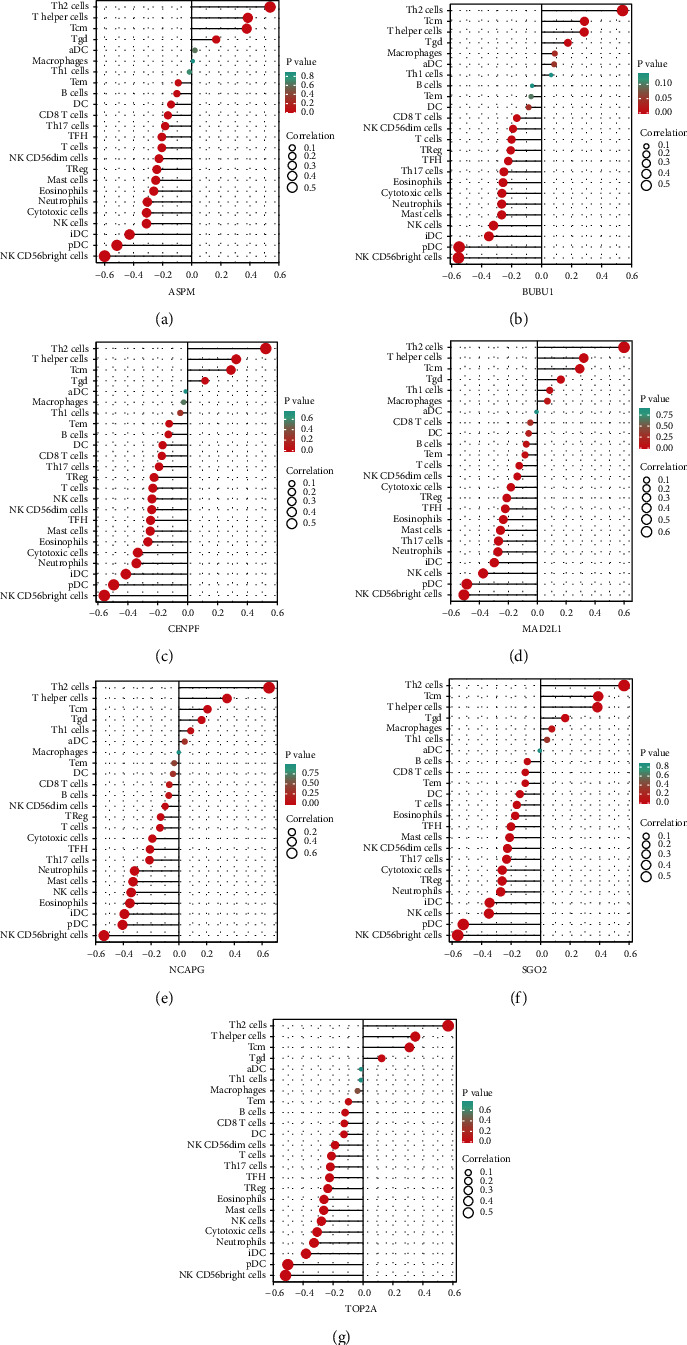
(a–g) Correlation of hub gene expression and immune cell infraction.

Finally, ssGSEA showed that *ASMP*, *BUB1*, *CENPF*, *MAD2L1*, *NCAPG*, *SGO2*, and *TOP2A* were positively related with the immune cells like Th2 cells (Figures 5(a)–5(g)). In contrast, all hub genes were negatively correlated with the immune cells, such as CD56 bright NK cells.

### 3.8. Assessment of UCEC Gene Expression

**Figure 6:**
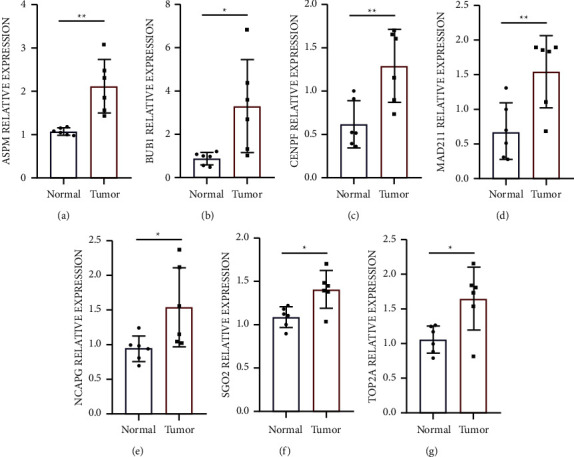
(a–g) The mRNA expression of hub genes in patients with UCEC via PCR.

To validate the expression of *ASMP*, *BUB1*, *CENPF*, *MAD2L1*, *NCAPG*, *SGO2*, and *TOP2A* genes in tumor samples and adjoining nontumor samples, we measured their relative mRNA expressions using qPCR. The findings illustrated that the average level of *ASMP*, *BUB1*, *CENPF*, *MAD2L1*, *NCAPG*, *SGO2*, and *TOP2A* expression was substantially elevated in UCEC tissues (Figures 6(a)–6(g)).

## 4. Discussion

With low incidence and mortality rates of UCEC, endometrial cancer often exhibits a favorable prognosis at an early stage [13]. However, at an advanced stage, it has poor prognosis, lacks specific early symptoms, and there are no effective strategies for early detection [14]. Developing a reliable prognostic model to offer parameters for determining clinical treatment choices is thus a priority [15].

DNA damage often occurs in cells under the pressure of exogenous agents, including exposure to ultraviolet (UV) light, ionizing radiation, and chemicals, as well as endogenous factors, such as replication errors and oxidative stress, which eventually cause single-strand breaks (SSBs) or double-strand breaks (DSBs) in DNA [16, 17]. DNA damage triggers a sophisticated signal transduction pathway that detects damage to DNA and transmits this data to the cell to trigger cellular responses to DNA damage. The DNA damage response maximizes the ability of the cells to repair DNA and increases their survival once the damage is done [18]. However, in severe DNA damage, apoptosis and cell senescence occur since DNA damage response halts advancement of the cell cycle [19]. The damage and repair machinery of DNA play a role in UCEC microsatellite instability, which is caused by HPV infection (which knocks out TP53) [20]. The DNA in a cell is capable of being remodeled and repaired due to an arsenal of enzymes that are present in the cell. Nonetheless, their actions must be strictly controlled in a spatial, temporal, and DNA lesion-appropriate manner to maximize DNA repair and minimize necessary and possibly harmful modifications in DNA structure throughout normal cellular functions. Cells have evolved to detect and rectify mistakes that occur during DNA replication so that they can appropriately preserve the integrity of the genetic code. If these mistakes cannot be fixed, mechanisms are put into place to either destroy the cell via a process called apoptosis and/or force the cell to go through a process called senescence. Nonetheless, one of the characteristics of cancer is genomic instability, which may be caused by interruption of the DNA damage response (DDR). This can contribute to the evasion of death and senescence, or the uncontrolled proliferation of cells with DNA replication errors.

Numerous research reports have demonstrated links between dysfunctional DNA repair pathways and malignancy, although most of these investigations are not associated with UCEC. One of the most significant benefits of our research is that it is an unbiased investigation transcending the candidate-gene method, and takes into consideration the complex interaction of DNA repair genes in a variety of UCECs. Meanwhile, some hub genes including *ASMP*, *BUB1*, *CENPF*, *MAD2L1*, *NCAPG*, *SGO2*, and *TOP2A* were found via bioinformatics analysis and experiment. Among these hub genes, *MAD2L1* has been reported to be associated with UCEC, and maintains the stemness characteristics of UCEC. Furthermore, *NCAPG* was confirmed to be dysregulated in UCEC in GSE63678 and GSE17025 [21]. Additional research ought to investigate the other hub genes to find more potential biomarkers for UCEC.

## 5. Conclusion

In this study, we used machine learning to identify distinct UCEC DNA damage-associated subtypes, each of which had distinct molecular properties, immunological features, and prognostic outcomes. Furthermore, hub genes were screened by bioinformatics analysis and confirmed by experiment. The microenvironment was analyzed by ssGSEA. In all, our findings may offer patients with UCEC a potential new treatment strategy.

## Figures and Tables

**Table 1:** Primer pairs for the target genes.

Gene	Forward primer sequence (5′-3′)	Reverse primer sequence (5′-3′)
ASPM	GCGTTTGCTTTTCAGGTGGA	CCTCCACATAGCCTGAATAAGTGA
BUB1	CGGCTTCTAGTTTGCGGTTC	ACCCACTGTATGTATTGAAGGAC
CENPF	CGTCCCCGAGAGCAAGTTTA	GTGGAAGAGTCTGGCTTGCT
MAD2L1	AGAGCCCAGGAGGAACTGAA	TGGATGGAGGCAACAAACGA
NCAPG	GGCGCCCATTGTTACTGTTG	AGCATCATTCTTCTCTATGTGGACT
SGO2	GAACCCAAAAATCAGGAATAGACC	ACTTCATCTTCTCATCTTGTCTCTG
TOP2A	CCGTCACCATGGAAGTGTCA	CATGTCTGCCACCCTTGGAT
GAPDH	AATGGGCAGCCGTTAGGAAA	GCCCAATACGACCAAATCAGAG

## Data Availability

All the experimental data involved in this study were obtained from open sources.

## Abbreviations

UCEC: Uterine corpus endometrial carcinoma
DEGs: Differentially expressed genes
TME: Tumor microenvironment
TCGA: The cancer genome atlas
FPKM: Fragments per kilobase million
TPM: Transcripts per million
GAPDH: Glyceraldehyde-3-phosphate dehydrogenase.

## Supplementary Materials

Supplementary Materials Table S1: DNA damage-related genes.
