# Supplementary material for: Novel DNA Damage-Related Subtypes Characterization Identifies Uterine Corpus Endometrial Carcinoma (UCEC) Based on Machine Learning
Source: J Oncol. 2022 Aug 28;2022:3588117. doi: 10.1155/2022/3588117 (PMC9441400; doi:10.1155/2022/3588117)
Supplement: Supplementary Materials — Table S1: DNA damage-related genes. [file 3588117.f1.docx]

**Table S1 DNA damage-related genes**

| **Gene symbol** | | | | | |
| --- | --- | --- | --- | --- | --- |
| RAD23A | UBE2B | UBE2A | POLM | XRCC6 | SETMAR |
| RAD17 | DCLRE1A | RNF8 | MMS19 | TREX1 | SLX1A |
| RMI2 | APLF | TELO2 | DDB2 | POLE | RAD23B |
| RAD54L | LIG3 | EME2 | ERCC2 | LIG4 | SPO11 |
| UBE2N | MUTYH | TDP2 | XRCC3 | FAN1 | RNASEH1 |
| MED1 | FANCD2 | PRIM1 | POLK | OGG1 | CETN2 |
| POLG | MGMT | RAD52 | MCM2 | GEN1 | FANCC |
| RECQL | NHEJ1 | MCM6 | HELQ | SLX1B | UBE2T |
| ENDOV | ATR | MCM7 | MSH2 | POLE2 | RPA4 |
| PRPF19 | APTX | POLD2 | DCLRE1B | POLN | MNAT1 |
| APEX2 | MLH3 | ECD | ATM | RFC3 | HAP1 |
| BRCA2 | DUT | MSH4 | POLH | MPG | ERCC1 |
| RPA3 | PMS2 | POLD1 | RFC2 | XRCC5 | RNH1 |
| RNASEH2B | XPC | CENPX | USP1 | HES1 | UVSSA |
| RAD18 | ALKBH2 | FANCF | RNF168 | HMGB1 | RAD9A |
| ATRIP | GTF2H5 | TOP3A | FANCL | RBBP8 | XRCC4 |
| ERCC8 | NTHL1 | NEIL3 | SEM1 | MRE11 | RNASEH2C |
| PRKDC | PARP3 | RECQL5 | CUL4A | MBD4 | SYCP3 |
| BRIP1 | CHAF1A | TOP3B | PCNA | POLD4 | FANCE |
| DDB1 | RBX1 | REV1 | MCM4 | XRCC2 | MSH3 |
| ERCC5 | TDP1 | RAD51C | PRIM2 | RNASEH2A | PMS1 |
| BIVM-ERCC5 | RAD51 | BLM | POLI | MUS81 | FAAP24 |
| POLQ | SHPRH | TREX2 | GTF2H3 | BACH1 | REV3L |
| CHEK1 | NBN | RNF4 | MDC1 | RFC1 | RAD51B |
| XPA | SPRTN | NEIL1 | PALB2 | FANCB | TOPBP1 |
| NUDT1 | POLD3 | TDG | POLB | POLE4 | DNTT |
| FANCG | CLK2 | FANCI | PNKP | HLTF | RRM2B |
| RFC5 | WRN | RIF1 | MCM3 | RAD50 | BRCA1 |
| ALKBH3 | RAD51D | PER1 | FAH | RPA1 | NEIL2 |
| POLA2 | HUS1 | RFC4 | RMI1 | ERCC3 | MSH6 |
| RDM1 | CUL4B | RAD54B | MCM5 | FEN1 | GTF2H2 |
| XRCC1 | XAB2 | RPA2 | ERCC4 | DNA2 | GTF2H2C |
| CCNL1 | PMS2CL | WDR48 | DMC1 | RAD1 | CCNH |
| FANCA | APEX1 | DCLRE1C | TP53 | CDK7 | UBE2V2 |
| LIG1 | SSBP1 | POLA1 | UNG | MAD2L2 | FAAP20 |
| MLH1 | TP53BP1 | SMUG1 | GTF2H4 | H2AFX | FAAP100 |
| CHEK2 | MSH5 | POLL | PARP1 | ERCC6 | POLE3 |
| PARP4 | FANCM | EXO1 | SLX4 | JUNB | PARP2 |
| EME1 | GTF2H1 | RECQL4 | CENPS |  |  |
